# Lack of Middle East Respiratory Syndrome Coronavirus Transmission in Rabbits

**DOI:** 10.3390/v11040381

**Published:** 2019-04-24

**Authors:** W. Widagdo, Nisreen M. A. Okba, Mathilde Richard, Dennis de Meulder, Theo M. Bestebroer, Pascal Lexmond, Elmoubasher A. B. A. Farag, Mohammed Al-Hajri, Koert J. Stittelaar, Leon de Waal, Geert van Amerongen, Judith M. A. van den Brand, Bart L. Haagmans, Sander Herfst

**Affiliations:** 1 Department of Viroscience, Erasmus Medical Center, 3015GD Rotterdam, The Netherlands; w.widagdo@erasmusmc.nl (W.W.); n.okba@erasmusmc.nl (N.M.A.O.); m.richard@erasmusmc.nl (M.R.); d.demeulder@erasmusmc.nl (D.d.M.); t.bestebroer@erasmusmc.nl (T.M.B.); p.lexmond@erasmusmc.nl (P.L.); j.m.a.vandenbrand@uu.nl (J.M.A.v.d.B.); s.herfst@erasmusmc.nl (S.H.); 2 Ministry of Public Health, Doha, Qatar, PO Box. 42; eabdfarag@MOPH.GOV.QA (E.A.B.A.F.); malhajri1@MOPH.GOV.QA (M.A.-H.); 3 Viroclinics Biosciences BV, Rotterdam 3029 AK, The Netherlands; stittelaar@viroclinics.com (K.J.S.); dewaal@viroclinics.com (L.d.W.); amerongen@viroclinics.com (G.v.A.)

**Keywords:** MERS-coronavirus, transmission, rabbits

## Abstract

Middle East respiratory syndrome coronavirus (MERS-CoV) transmission from dromedaries to humans has resulted in major outbreaks in the Middle East. Although some other livestock animal species have been shown to be susceptible to MERS-CoV, it is not fully understood why the spread of the virus in these animal species has not been observed in the field. In this study, we used rabbits to further characterize the transmission potential of MERS-CoV. In line with the presence of MERS-CoV receptor in the rabbit nasal epithelium, high levels of viral RNA were shed from the nose following virus inoculation. However, unlike MERS-CoV-infected dromedaries, these rabbits did not develop clinical manifestations including nasal discharge and did shed only limited amounts of infectious virus from the nose. Consistently, no transmission by contact or airborne routes was observed in rabbits. Our data indicate that despite relatively high viral RNA levels produced, low levels of infectious virus are excreted in the upper respiratory tract of rabbits as compared to dromedary camels, thus resulting in a lack of viral transmission.

## 1. Introduction

Middle East respiratory syndrome coronavirus (MERS-CoV) is a novel pathogen that is known to infect dromedary camels and humans [1,2]. Seroepidemiological studies indicate that this virus has been circulating in dromedary camels in the Arabian Peninsula and Africa for decades [3,4,5]. MERS-CoV sequences obtained from these camels are largely similar to those obtained from human MERS cases in corresponding regions, thus providing evidence that dromedary camels act as the zoonotic source of this virus [6,7]. However, many primary human MERS cases do not have a history of direct contact with these animals [8]. This suggests the presence of unidentified routes of human-to-human transmission or the involvement of other animal species in spreading the virus to humans. Besides dromedary camels, other animal species, i.e. llamas, alpacas, and pigs have been shown to be susceptible and develop upper respiratory tract infection upon experimental intranasal MERS-CoV inoculation [9,10,11]. This is in line with the expression of the MERS-CoV receptor, dipeptidyl peptidase-4 (DPP4), in their nasal epithelium [9]. MERS-CoV-seropositive llamas and alpacas have been reported in the field, and MERS-CoV-experimentally-inoculated alpacas have also been shown to transmit the virus via contact [12,13,14].

To further understand the zoonotic potential of MERS-CoV, it is crucial to delineate the factors involved in the spread of the virus among dromedaries as well as other animal species. In order to gain insight into these factors, we performed MERS-CoV transmission experiments in rabbits. We have previously shown that rabbits are susceptible to MERS-CoV and develop both upper and lower respiratory tract infection upon virus inoculation [15]. Naïve recipient rabbits were housed with MERS-CoV-inoculated donor rabbits either in the same or in adjacent cages to determine whether MERS-CoV can be transmitted via contact or airborne routes, respectively [16]. Donor rabbits were found to shed high levels of viral RNA but a limited amount of infectious virus thus potentially restricting MERS-CoV transmission in these animals.

## 2. Materials and Methods

### 2.1. Virus Stocks

In vivo experiments in this study were performed using Passage 7 human isolate MERS-CoV EMC strain (HCoV-EMC/2012) and passage 3 isolate MERS-CoV (Qatar15/2015; GenBank Acc. No. MK280984) that were propagated in Vero cells as described earlier [9]. Qatar15 was isolated from a 69 years old Qatari man that developed severe pneumonia and was PCR confirmed to have a MERS-CoV infection [17].

### 2.2. Animal Experiments

Animal experiments were approved and performed according to the guidelines from the Institutional Animal Welfare Committee (no. 201300121 approved on 17 July 2013, 122-17-01 approved on 28 September 2017, and AVD277002015283-WP01 approved on 2 November 2016). The studies were performed under biosafety level 3 (BSL3) conditions. To compare whether different routes of MERS-CoV inoculation generate similar clinical outcomes, twelve 6-month-old New Zealand rabbits (*Oryctolagus cuniculus*), specific pathogen free, and seronegative for MERS-CoV were divided into four groups. Animals were inoculated under ketamine-medetomidine anesthesia either (a) intranasally with 200 µL of 1 × 10^6^ TCID_50_/mL MERS-CoV; (b) intranasally with 1 mL 1 × 10^6^ TCID_50_/mL MERS-CoV; (c) intranasally with 200 µL of 1 × 10^6^ TCID_50_/mL MERS-CoV and intratracheally with 3 mL 4 × 10^6^ TCID_50_/mL MERS-CoV; or (d) intranasally with 1 mL PBS. The intranasal inoculums were divided equally over both nostrils. Nasal and throat swabs were obtained daily from day 1 up to day 4 post inoculation. These animals were then sacrificed on day 4 and respiratory tract tissues were collected. To compare the clinical outcomes of the MERS-CoV EMC strain and the Qatar15 strain, ten New Zealand rabbits were divided into two groups and inoculated with 1 mL of 1 × 10^6^ TCID_50_/mL of each MERS-CoV strain intranasally. Nasal and throat swabs were obtained from day 1 up to day 4 post inoculation and these animals were sacrificed on day 4.

To study MERS-CoV transmission, a modified version of the previously described influenza A virus ferret transmission set-up was used. This set-up consists of two clear polymethyl methacrylate cages of different sizes. Donor rabbits and direct contact recipients were housed in a cage of 35 cm × 30 cm × 65 cm (W × H × L), whereas airborne recipients were housed in a cage of 30 cm × 30 cm × 55 cm (W × H × L). These cages are separated by two stainless steel grids 10 cm apart to prevent direct contact but still allow airflow from the donor rabbit to the airborne recipient rabbit. These transmission cages allow the experiment to be conducted in negatively pressured isolators in the BSL3 facility, with HEPA-filtered airflow <0.1 m/s [18]. Since these cages were too small for New Zealand rabbits, we chose a smaller-sized breed, the Netherland dwarf rabbits (*Oryctolagus cuniculus*), for the MERS-CoV transmission experiment. We used both male and female rabbits with an age range of 6 months–3 years in these experiments. First, three MERS-CoV seronegative Netherland dwarf rabbits were inoculated intranasally with 1 mL of 1 × 10^6^ TCID_50_/mL MERS-CoV Qatar15 strain (500 µL per nostril) to show that they are equally susceptible to MERS-CoV as the New Zealand rabbits. Nasal and throat swabs were obtained daily up to 4 days post inoculation. These animals were then sacrificed on day 4 and their respiratory tract tissues were collected. For the virus transmission experiment, twelve Netherland dwarf rabbits were randomly distributed into four individually housed groups. One naïve rabbit from each group was inoculated intranasally with 1 mL of 1 × 10^6^ TCID_50_/mL MERS-CoV (500 µL per nostril), thus acting as donor rabbits. The other two rabbits were used as direct contact and airborne recipients, respectively. Donor and direct contact animals were of the same sex. All animals were sacrificed at 14 days post exposure and blood was collected to assess seroconversion. 

### 2.3. Virological Analysis

Nasal swabs, throat swabs, and respiratory tract tissue samples were evaluated for the presence of infectious virus by virus titration, and for viral RNA by RT-qPCR against the UpE gene as previously described [9]. Samples with a cycle threshold less than forty were considered as positive for MERS-CoV RNA. Viral RNA was quantified as genome equivalents (GE) using MERS-CoV strain EMC (containing 10^6^ TCID_50_/mL) as a calibrator. Virus titration was performed in serial 10-fold dilutions on Vero cells. Cells were monitored under a light microscope at day 6 for cytopathic effect. The amount of infectious virus in swab samples was calculated by determining the TCID_50_. Statistical analysis was performed using the GraphPad Prism program (La Jolla, CA, USA). Kruskal–Wallis test was applied due to the non-normal distribution of our data as priorly determined by Shapiro–Wilk test. The significant difference between groups was determined at a *P*-value < 0.05.

### 2.4. Histopathology and Histochemistry Analysis

Respiratory tract tissue samples were collected in formalin and embedded in paraffin for pathological analysis. Hematoxylin-eosin staining was performed for histopathological analysis. The presence of MERS-CoV nucleoprotein and MERS-CoV RNA was detected by immunohistochemistry and in-situ hybridization, respectively, using previously published protocols [15]. The localization of DPP4 in the respiratory tract of non-infected New Zealand rabbits was analyzed using an optimized immunohistochemical assay [15,19].

### 2.5. Serological Analysis

Collected serum samples were tested for MERS-CoV neutralizing antibodies using a virus neutralization assay and for MERS-CoV S1-specific antibodies using MERS-CoV S1 ELISA according to the previously published protocols [9]. Goat anti-rabbit IgG conjugated with HRP (1:2000, DAKO, Glostrup, Denmark) was used as a secondary antibody in the ELISA.

## 3. Results

### 3.1. Dipeptidyl peptidase-4 DPP4 is Expressed in the Upper and Lower Respiratory Tract of Rabbits

**Figure 1:**
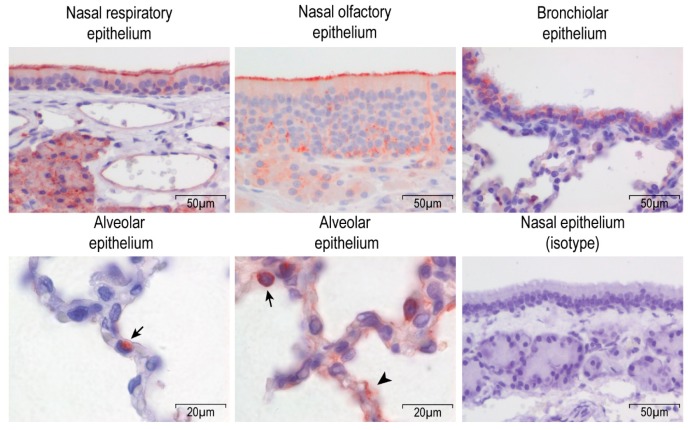
Dipeptidyl peptidase-1 (DPP4) expression in the respiratory tract tissues of rabbits. DPP4 is detected using immunohistochemistry and indicated in red in the figure. Type II cells are indicated with arrows, and type I cells with an arrowhead. Nasal epithelium and bronchiolar epithelium pictures were taken at a 400× magnification, and alveolar epithelium at 1000×. The isotype control showed no background signal in our assay.

Rabbits are the smallest animal species that can be naturally infected by MERS-CoV. We previously reported that they develop both upper and lower respiratory tract infection upon MERS-CoV inoculation [15], suggesting the expression of the viral receptor at these locations. Using immunohistochemistry, we analyzed the DPP4 expression in rabbit respiratory tract tissues. In the upper respiratory tract, DPP4 is strongly expressed at the apical surface of both nasal respiratory and olfactory epithelium (Figure 1). In the lower respiratory tract, DPP4 is present in both bronchiolar and alveolar epithelial cells, although some variation in DPP4 expression was observed throughout the lungs. DPP4 is either absent, limitedly expressed on alveolar type II cells, or expressed on both alveolar type I and II cells (Figure 1). Thus, these data highlight a broad DPP4 expression in the respiratory tract tissues of rabbits. Our results show that rabbits express DPP4 in both the upper and lower respiratory tract epithelium, in line with MERS-CoV tropism in this species [9,19].

### 3.2. Middle East Respiratory Syndrome Coronavirus (MERS-CoV) Infects Both Upper and Lower Respiratory Tract of Rabbits upon Intranasal Inoculation

**Figure 2:**
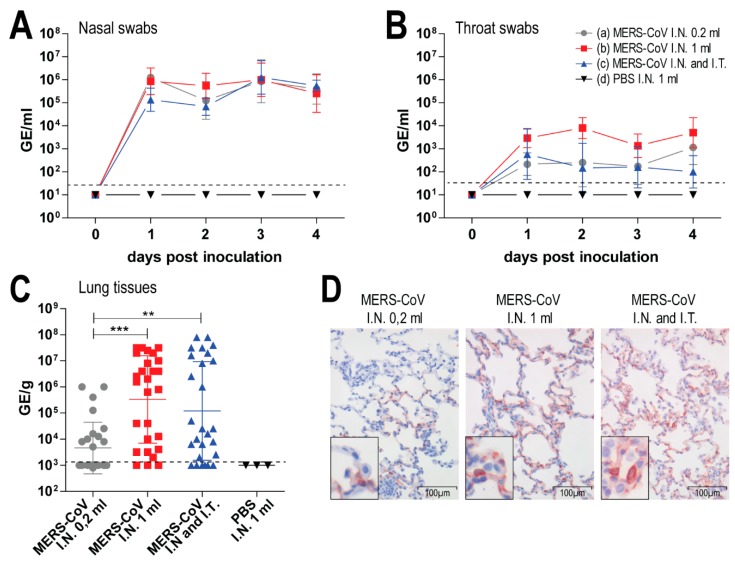
Middle East respiratory syndrome coronavirus (MERS-CoV) inoculation in rabbits with different routes and volumes. Three New Zealand rabbits were each infected either with (a) 200 µL of 1 × 10^6^ TCID_50_/mL MERS-CoV intranasal (I.N.); (b) 1 mL of 1 × 10^6^ TCID_50_/mL MERS-CoV I.N.; (c) 200 µL of 1 × 10^6^ TCID_50_/mL MERS-CoV I.N. combined with 3 mL of 4 × 10^6^ TCID_50_/mL MERS-CoV intratracheal (I.T.); or (d) 1mL of PBS I.N as negative control. These animals were sacrificed at day 4 post inoculation. Viral RNA shed by the MERS-CoV-inoculated rabbits in the nasal swabs (**A**) and throat swabs (**B**) are reported in genome equivalents per ml (GE/mL). Viral RNA detected in the lungs of these rabbits are reported in genome equivalents per gram tissues (GE/g). Dashed lines depict the detection limit of the assays. All error bars represent standard deviations. Statistical analysis is performed using Kruskal wallis test (**, *p* value < 0.01; ***, *p* value < 0.001). Representative figures of immunohistochemistry detecting MERS-CoV nucleoprotein (displayed in red) in the lungs of these rabbits were taken at a 200× magnification (**D**). All MERS-CoV-inoculated rabbits shed a relatively equal amount of viral RNA in the nasal and throat swabs (**A**,**B**). However, there was significantly less viral RNA in the lungs of rabbits in the group (a) in comparison to the other MERS-CoV-inoculated groups (**C**). A similar finding is observed in our immunohistochemistry analysis detecting MERS-CoV nucleoprotein (displayed in red) in the lungs of these rabbits (**D**). Pictures were taken in 200× magnification. The amount of viral RNA is displayed either in genome equivalent per mL (GE/mL) or per gram tissues (GE/g). Dashed lines depict the detection limit of the assays. All error bars represent standard deviations. Statistical analysis is performed using Kruskal–Wallis test (**, *p* value < 0.01; ***, *p* value < 0.001).

In our previous study, we inoculated rabbits both intranasally and intratracheally [15]. Intratracheal inoculation is quite invasive, and thus requires a skillful operator to minimize procedure-related damage in the respiratory tract. In contrast, intranasal inoculation is less invasive and had been used in other studies to infect rabbits with MERS-CoV [20,21]. Here we investigated whether intranasal MERS-CoV inoculation is sufficient to induce both upper and lower respiratory tract infection in rabbits, in comparison to combined intranasal and intratracheal inoculation. Three New Zealand rabbits (*Oryctolagus cuniculus*) were inoculated with MERS-CoV EMC strain either intranasally with 200 µL of 1 × 10^6^ TCID_50_/mL (group a); intranasally with 1 mL of 1 × 10^6^ TCID_50_/mL (group b); intranasally with 200 µL of 1 × 10^6^ TCID_50_/mL and intratracheally with 3 mL of 4 × 10^6^ TCID_50_/mL (group c); or intranasally with 1ml of PBS as a negative control (group d). All three groups of MERS-CoV inoculated rabbits developed minimal clinical manifestations and histopathological lesions. The amount of viral RNA shed in the nasal and throat swabs did not vary among the inoculated groups (Figure 2A,B). However, in the lungs of the rabbits, the amount of viral RNA was significantly lower in group a than in groups b and c (Figure 2C). In line with these observations, fewer MERS-CoV-infected cells were observed in the lungs of group a animals compared to groups b and c (Figure 2D). Based on these results, we decided to use intranasal inoculation with 1 mL of 1 × 10^6^ TCID_50_/mL MERS-CoV for our subsequent experiments.

**Figure 3:**
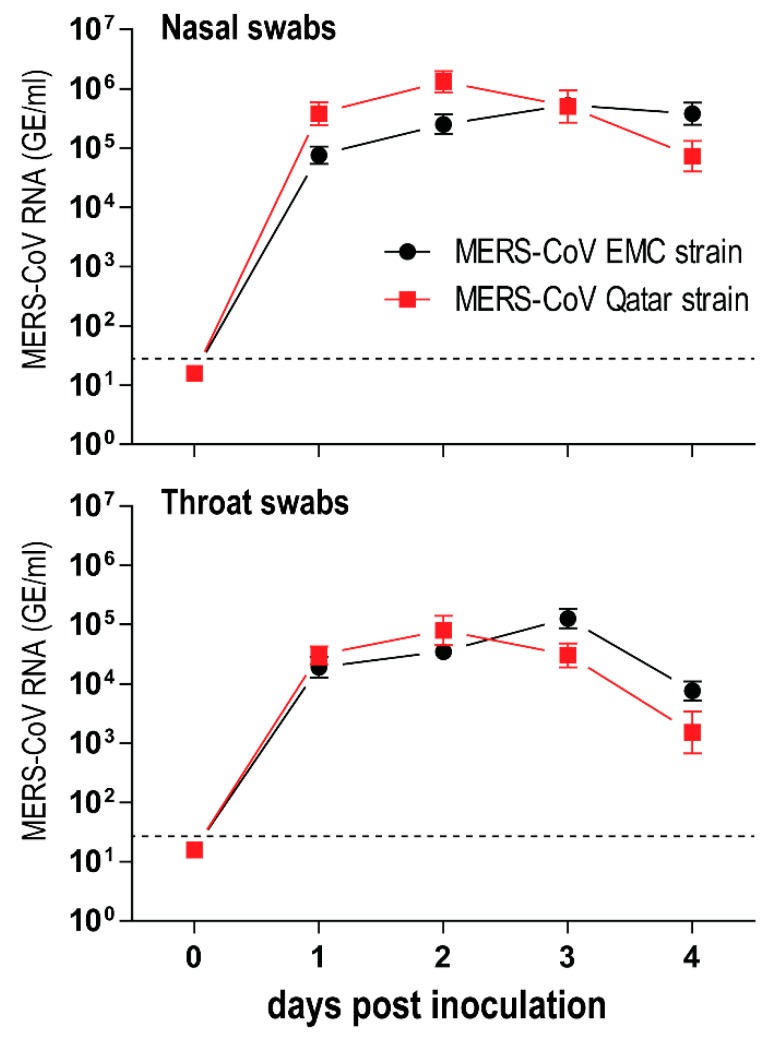
Middle East respiratory syndrome coronavirus (MERS-CoV) EMC strain and Qatar15 strain replicate equally in the upper respiratory tract of rabbits. Five New Zealand rabbits were each intranasally inoculated either with 1 mL of 1 × 10^6^ TCID_50_/mL MERS-CoV EMC strain or Qatar15 strain. Nasal and throat swabs were obtained from day 0 (before inoculation) until day 4 post inoculation. The amount of viral RNA is displayed in genome equivalents per mL (GE/mL). Dashed lines depict the detection limit of the assay. All error bars represent standard deviations.

Different human MERS-CoV strains have been isolated since the EMC/2012 strain was first characterized [22,23]. However, studies that evaluate phenotypic differences between these strains in animals are currently lacking. We investigated whether a more recent MERS-CoV strain (Qatar15/2015) replicates differently in rabbits in comparison to the EMC strain. We found that rabbits inoculated with the MERS-CoV EMC strain and those with the Qatar15 strain developed an equally mild infection and shed similar levels of viral RNA in their nasal and throat swabs (Figure 3). Following this result, the MERS-CoV transmission experiment was performed using the Qatar15 strain, the more recent strain of these two. 

### 3.3. Middle East Respiratory Syndrome Coronavirus (MERS-CoV) Transmission to Contact and Airborne-Exposed Rabbits

**Figure 4:**
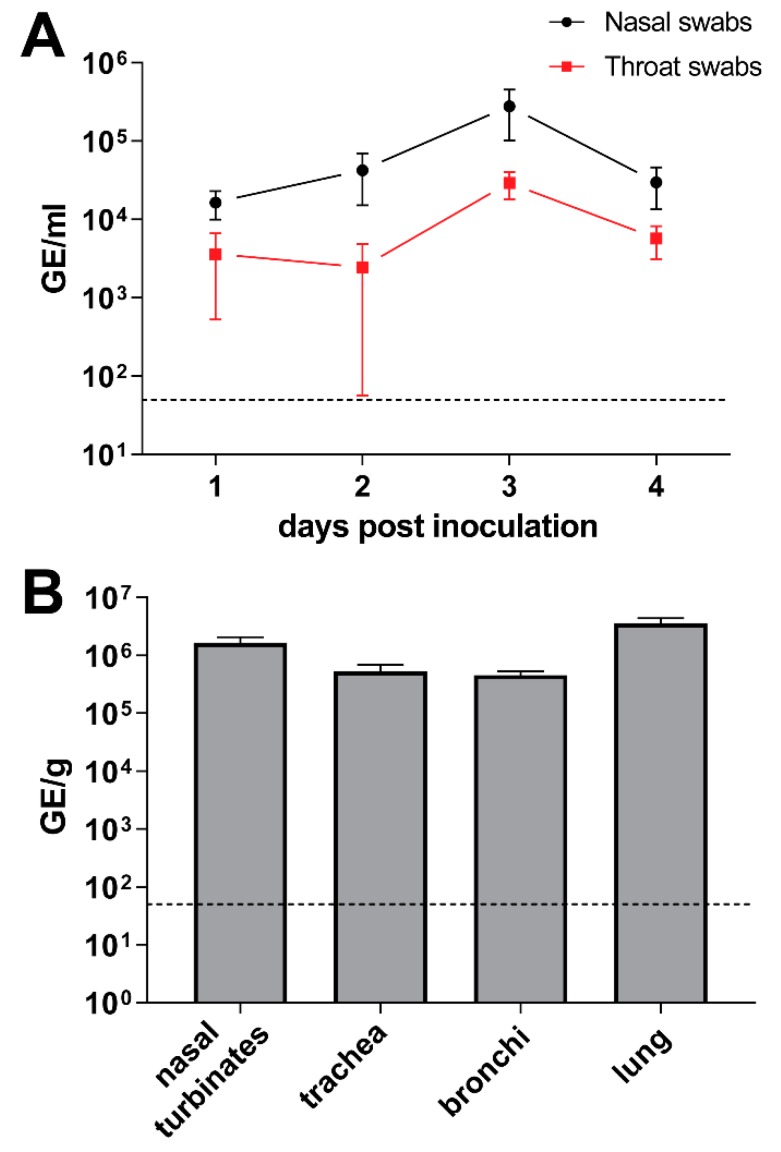
Middle East respiratory syndrome coronavirus (MERS-CoV) infects the upper and lower respiratory tract of Netherland dwarf rabbits. The amount of viral RNA in the nasal and throat swabs (**A**) as well as in the respiratory tract tissues (**B**) are displayed in genome equivalents per mL (GE/mL) and genome equivalents per gram tissues (GE/g), respectively. Dashed lines depict the detection limit of the assays. All error bars represent standard error of means.

To study MERS-CoV transmission, an experimental set up previously used to investigate influenza A virus transmission between ferrets was used. This set up consists of two polymethyl methacrylate cages separated with two steel grids, 10 cm apart [16]. Because this set-up was too small to house New Zealand rabbits, we used a smaller-sized breed, the Netherland dwarf rabbits. Prior to the virus transmission experiment, Netherland dwarf rabbits were inoculated with MERS-CoV Qatar 15 strain to determine their susceptibility to the virus. Similar to the New Zealand rabbits [15], Netherland dwarf rabbits shed viral RNA in the nasal and throat swabs as well as in the respiratory tract tissues upon intranasal inoculation (Figure 4A,B). Identical to the New Zealand rabbits [15], the MERS-CoV-inoculated Netherland dwarf rabbits did not develop any clinical signs, including nasal discharge, and showed minimal histopathological lesions and immune cell infiltration in the respiratory tract.

**Figure 5:**
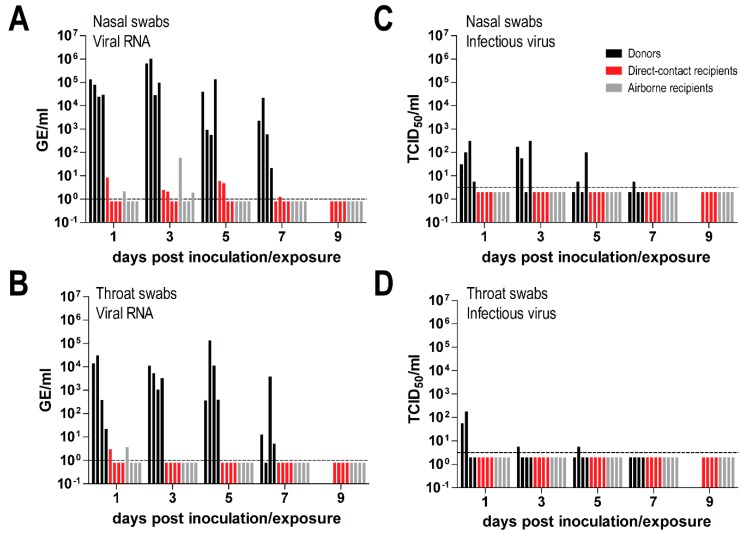
Shedding of Middle East respiratory syndrome coronavirus (MERS-CoV) RNA and infectious virus in directly inoculated, contact-exposed and airborne-exposed rabbits. MERS-CoV RNA and infectious virus were measured in the nasal (**A**,**B**) and throat swabs (**C**,**D**). The amount of viral RNA is displayed in genome equivalents per mL (GE/mL), while infectious virus is shown as 50% tissue culture infective dose per ml (TCID_50_/mL). Dashed lines depict the detection limit of each assay.

**Figure 6:**
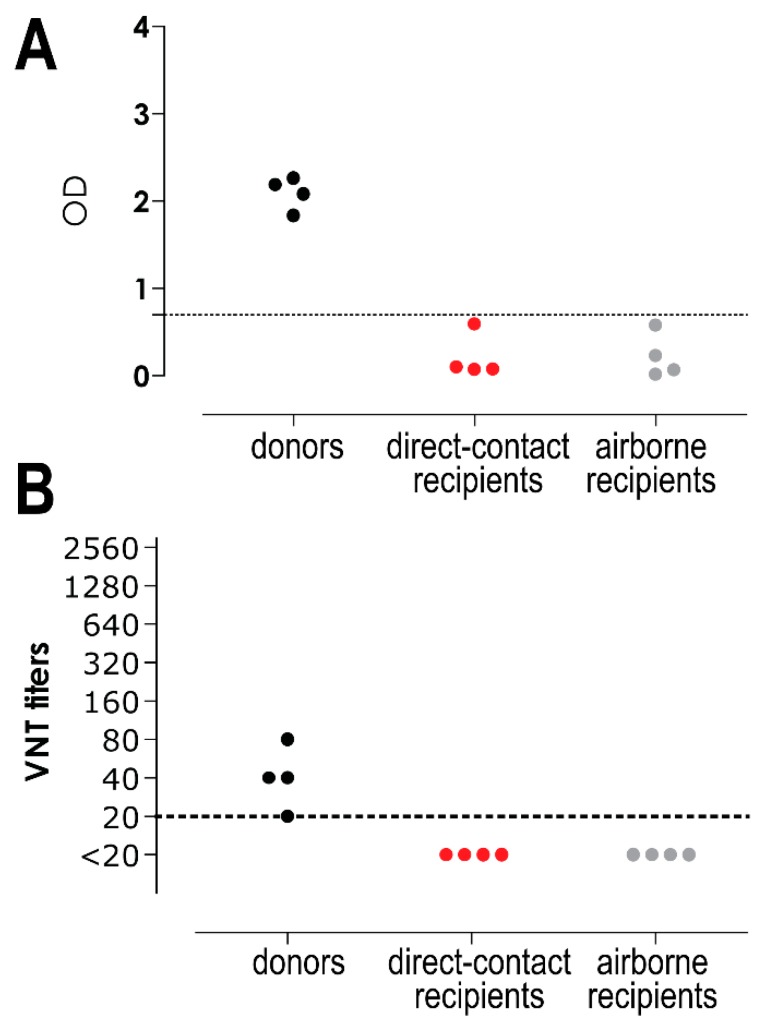
Middle East respiratory syndrome coronavirus (MERS-CoV)-specific antibody response in rabbits. S1-specific MERS-CoV antibodies were measured with ELISA and displayed as optical density (OD) value (**A**), while MERS-CoV neutralizing antibodies were measured with the virus neutralization test (VNT) and displayed in titers (**B**). Dashed lines depict the detection limit of the assay.

To study MERS-CoV transmission, four Netherland dwarf rabbits were intranasally inoculated with MERS-CoV. Six-hours later, each of them was co-housed with one naïve rabbit in the same cage, and 24 h later another one was co-housed in an adjacent cage to determine whether MERS-CoV could be transmitted through contact and/or airborne routes. Nasal and throat swabs were collected every other day up to day 7 or 9 post inoculation/exposure for the donor and direct contact rabbits, respectively, and day 9 post exposure for the airborne recipient ones. Both viral RNA and infectious virus were quantified in these samples. We found that all donor rabbits shed high loads of viral RNA in both the nasal swabs (~10^5^–10^6^ TCID_50_ GE/mL) and the throat swabs (~10^3^–10^4^ TCID_50_ GE/mL). The amount of viral RNA shed by the inoculated rabbits remained high until day 7 post inoculation. On the other hand, recipient rabbits housed in the same cage (direct contact recipients), or adjacent cage (airborne recipients), shed limited amounts of viral RNA (~10 TCID_50_ GE/mL) in both nasal and throat swabs. Among four animals in each group, only two direct contact recipient and two airborne recipient rabbits had detectable viral RNA up to day 5 post inoculation in the nasal swabs, while in the throat swabs, viral RNA was only detected in one direct contact recipient and one airborne recipient rabbit at day 1 post inoculation (Figure 5A,B). Infectious virus was detected at low level (~10^2^ TCID_50_/mL) both in the nasal and throat swabs of the donor rabbits; in the nasal swabs of all donor rabbits at day 1 post inoculation, and in one of the donors up to day 7. In the throat swabs, infectious virus was only detected in two donors on day 1, up to day 5 in one of them. In contrast, none of the swabs from recipient rabbits was positive for virus titration (Figure 5C,D). Serological analysis of samples collected 14 days after exposure showed that only the donor rabbits seroconverted and developed neutralizing antibodies (Figure 6A,B). The antibody response of these directly inoculated rabbits was relatively low, confirming the results of previous studies [15,21]. This indicates that these rabbits developed MERS-CoV infection while the contact and airborne-exposed rabbits did not, supporting the results of the virus titration. 

## 4. Discussion

Current data indicate that MERS-CoV is highly endemic in dromedary camels in the Arabian Peninsula and Africa and has been circulating in these animals for decades [3,4,5,24]. This suggests that this virus is easily transmitted between dromedary camels. From an epidemiological point of view, it is important to know whether other animal species in the region may also spread the virus to humans or other animal species. In vitro, MERS-CoV has been found to infect cells from a broad range of animal species including Old and New World camelids as well as primates, bats, cows, sheep, goats, pigs, horses, and rabbits [15,25,26]. The DPP4 viral receptor of these species, especially rabbits, has high similarity to that of humans and dromedary camels, especially in the region that interacts with the spike protein, and thus can facilitate MERS-CoV infection [25,26,27]. The New World camelids, i.e. llamas and alpacas, have been shown to seroconvert to MERS-CoV when present in regions where MERS-CoV is circulating and may transmit the virus [12,13,14]. It is currently unclear why, besides camelids, other livestock animals do not seem to transmit the virus to humans [24,28,29,30]. To further understand the transmission potential of MERS-CoV, we performed virus transmission experiments using rabbits as animal model.

To perform the virus transmission experiments, we housed MERS-CoV-inoculated rabbits together with naïve contact rabbits either in the same or adjacent cages. Rabbits developed both upper and lower respiratory tract infection upon MERS-CoV inoculation [15], either via intranasal or combined intranasal and intratracheal routes, in line with the localization of DPP4 in their respiratory tract epithelium. The amount of viral RNA being shed by the inoculated rabbits during the first three days post inoculation is almost as high as that of the MERS-CoV-inoculated dromedary camels [31,32]. However, none of the direct contact and airborne-exposed rabbits developed any clinical signs, shed significant levels of viral RNA, shed infectious virus, nor did they seroconvert. One possible reason for this lack of transmission is the limited amount of infectious virus being shed by the inoculated rabbits [21]. Previous studies have shown that in the nasal and lung tissues of experimentally infected rabbits, infectious virus was generally detected in a limited amount despite the abundant presence of viral RNA and virus nucleoprotein [15,21]. Alternatively, low levels of infectious virus transmitted to recipient animals may be unable to initiate a productive infection due to the presence of host proteins that restrict replication. Comparable to rabbits, MERS-CoV-infected pigs and goats develop minimal clinical signs, hardly shed infectious virus, and barely spread the virus to naïve animals [28,33]. In contrast, MERS-CoV-infected dromedary camels develop nasal discharge and shed a high amount infectious virus (10^4^–10^5^ TCID_50_/mL), almost equal to the amount of viral RNA being shed (10^5^–10^6^ GE/mL), during the first 4 days post inoculation [31,32]. These interspecies differences may indicate presence of nasal discharge and infectious virus shedding as critical factors in MERS-CoV transmission. In humans, levels of infectious virus shed by MERS-CoV patients have rarely been reported. However, MERS-CoV patients that transmit the virus have been shown to shed a higher amount of viral RNA in their swabs compared to those that do not, supporting the quantity of virus shed as an important factor in the transmission of MERS-CoV between humans [34]. For influenza A viruses, infectious virus shedding has been documented as one of the main determinants of airborne virus transmission. Using ferrets as an animal model, it has been reported that a reduction in infectious virus shedding in the nasal swabs can subsequently limit virus transmission [35,36,37]. 

Our results show that despite the presence of DPP4 in the upper respiratory tract, accompanied by MERS-CoV replication at this site, a limited amount of infectious virus was shed. Similarly, titration of rabbit lung homogenates that show high levels of viral RNA and presence of nucleoprotein (Figure 2C,D) resulted in only low levels of infectious virus, in line with earlier observations [15,21]. At this stage, it is not clear which host mediated mechanisms limit the production of infectious virus while allowing viral RNA to still be shed at high levels. Since restriction of infectious virus shedding in the rabbits already occurred one day post inoculation, activation of host innate immune responses, including type I interferon induction, may be relevant. In vitro studies have shown that MERS-CoV is relatively sensitive to type I interferon-mediated antiviral activities [38,39]. In human plasmacytoid dendritic cells, MERS-CoV inoculation leads to secretion of large amount of type I and III interferons and production of viral RNA, but hardly any infectious virus is being produced [40]. It is also possible that most infectious virus shed by these rabbits is defective, lacking the capacity to efficiently infect target cells. Further studies are needed to elucidate the mechanisms that restrict MERS-CoV replication in rabbits compared to dromedary camels. Potentially, some of the MERS-CoV accessory proteins shown to antagonize immune responses including production of interferon, may not work efficiently in some MERS-CoV susceptible species, including rabbits. It is intriguing to investigate whether a similar phenomenon occurs in some human MERS-CoV infections and whether this is linked to the development of asymptomatic to mild clinical manifestations [41]. This might partly explain why MERS-CoV transmission in humans is rather inefficient in comparison to dromedary camels [42,43], and why camelids that secrete high levels of infectious virus are the only known zoonotic source of MERS-CoV [2,7,14,31]. Deciphering these mechanisms could potentially offer insight into understanding MERS-CoV transmission as well as developing novel treatments to tackle the ongoing outbreaks.
